# Supplementary material for: 8–17 DNAzyme Silencing Gene Expression in Cells via Cleavage and Antisense
Source: Molecules. 2022 Dec 29;28(1):286. doi: 10.3390/molecules28010286 (PMC9821912; doi:10.3390/molecules28010286)
Supplement: Supplementary file 1 [file molecules-28-00286-s001.zip › molecules-2143439-supplementary.pdf]

*Supporting Material*

# 8–17 DNAzyme Silencing Gene Expression in Cells via Cleavage and Antisense

**Zhongchun Zhou [1], Wen Sun [1] and Zhen Huang [1,2,*]**

[1] Key Laboratory of Bio-Resource and Eco-environment of Ministry of Education, College of Life Sciences, Sichuan University, Chengdu 610064, China

[2] SeNA Research Institute & Szostak-CDHT Large Nucleic Acids Institute, Chengdu 610041, China

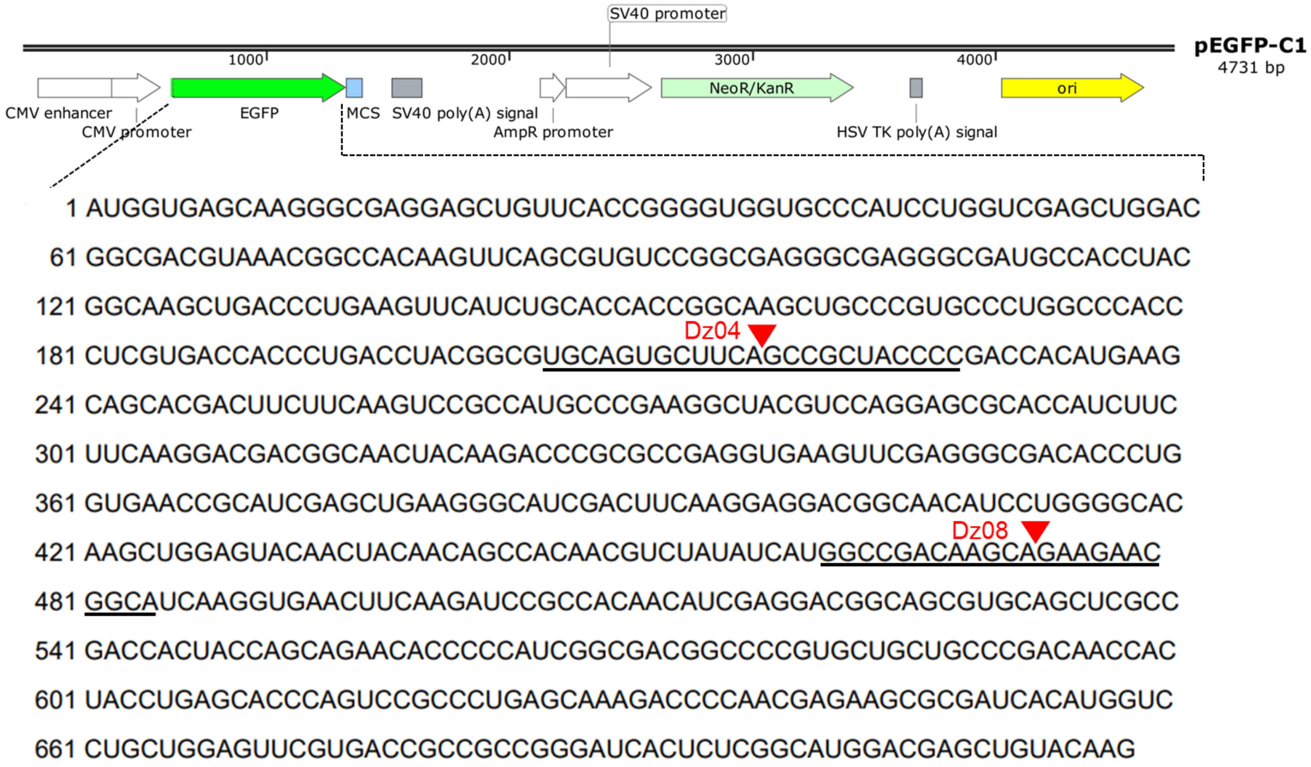


**Figure S1.** EGFP mRNA sequence and the target sites of Dz04 and Dz08.

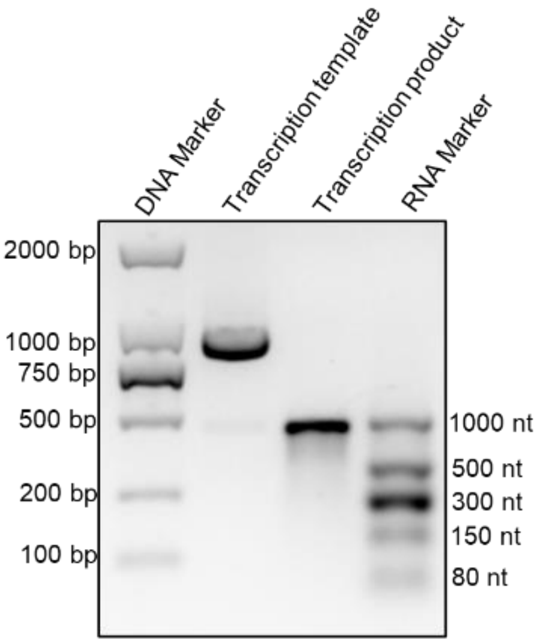


**Figure S2.** Agarose gel analysis of EGFP mRNA by *in vitro* transcription.

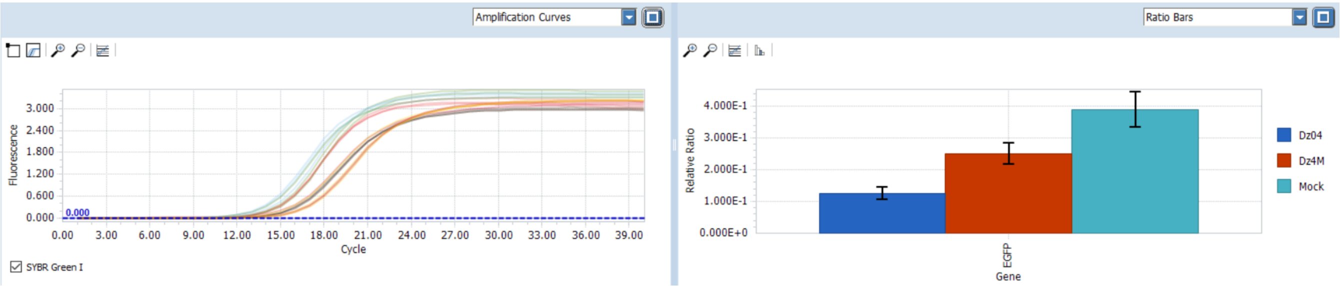


**Figure S3.** The original real time PCR trace of a typical experiment in Figure 4D.

**Table S1.** The sequences of short RNA substrates.

| Name | Sequence (5'-3') |
|---|---|
| Short RNA substrate (Dz04) | FAM-UGCAGUGCUUCAGCCGCUACCCC |
| Short RNA substrate (Dz08) | FAM-GGCCGACAAGCAGAAGAACGGCA |

**Table S2.** The sequences of Dz04, Dz4M, Dz4S and mismatched controls.

| Dz8-17 | Sequence (5'-3')[a] |
|---|---|
| Dz04 | GsGsGGTAGCGGTCCGAGCCGGTCGAAGAAGCACTGsCsA |
| Dz4M[b] | GsGsGGTAGCGG***C***CCGAGCCGGTCGAAGAAGCACTGsCsA |
| Dz4S[c] | GsGsAGGGCGTGTCCGAGCCGGTCGAATGACAGACAsGsC |
| Dz04-L2[d] | GsGsG***A***TAG***T***GGTCCGAGCCGGTCGAAGAAGCACTGsCsA |
| Dz04-R2[d] | GsGsGGTAGCGGTCCGAGCCGGTCGAAGAA***A***CAC***C***GsCsA |
| Dz04-L2/R2[d] | GsGsG***A***TAG***T***GGTCCGAGCCGGTCGAAGAA***A***CAC***C***GsCsA |

(**a**) Red fond, catalytic core sequence of Dz8-17; Italic bold, mutant bases; s, PS; (**b**) Dz04 mutant. [c] Sequence-scrambled control; [d] Dz04 with two mismatches in the left arm (L2) or right arm (R2) or both (L2/R2).

**Table S3.** The sequences of Dz04 and Scr control with different modifications at both arms.

| Name | Sequence (5'-3')[a] |
|---|---|
| Dz04 | GGGGTAGCGGTCCGAGCCGGTCGAAGAAGCACTGCA |
| Dz04-4s[b] | G$_s$G$_s$GGTAGCGGTCCGAGCCGGTCGAAGAAGCACTG$_s$C$_s$A |
| Dz04-4OMe[b] | GGGGTAGCGGTCCGAGCCGGTCGAAGAAGCACTGCA |
| Dz4S-4OMe[c] | GGAGGGCGTGTCCGAGCCGGTCGAATGACAGACAGC |
| Dz04-4s(2OMe)$_m$[b] | G$_s$G$_s$GGTAGCGGTCCGAGCCGGTCGAAGAAGCACTG$_s$C$_s$A |
| Dz4S-4s(2OMe)$_m$[c] | G$_s$G$_s$AGGGCGTGTCCGAGCCGGTCGAATGACAGACA$_s$G$_s$C |
| Dz04-4s(4OMe)$_m$[b] | G$_s$G$_s$GGTAGCGGTCCGAGCCGGTCGAAGAAGCACTG$_s$C$_s$A |
| Dz4S-4s(4OMe)$_m$[c] | G$_s$G$_s$AGGGCGTGTCCGAGCCGGTCGAATGACAGACA$_s$G$_s$C |
| Dz04-4s(6OMe)$_m$[b] | G$_s$G$_s$GGTAGCGGTCCGAGCCGGTCGAAGAAGCACTG$_s$C$_s$A |
| Dz04-4LNA[b] | GGGGTAGCGGTCCGAGCCGGTCGAAGAAGCACTGCA |
| Dz4M-4LNA[d] | GGGGTAGCGGCCCGAGCCGGTCGAAGAAGCACTGCA |
| Dz4S-4LNA[c] | GGAGGGCGTGTCCGAGCCGGTCGAATGACAGACAGC |
| Dz04-4s(2LNA)$_m$[b] | G$_s$G$_s$GGTAGCGGTCCGAGCCGGTCGAAGAAGCACTG$_s$C$_s$A |
| Dz4S-4s(2LNA)$_m$[c] | G$_s$G$_s$AGGGCGTGTCCGAGCCGGTCGAATGACAGACA$_s$G$_s$C |
| Short RNA substrate (Dz04) | FAM-UGCAGUGCUUCAGCCGCUACCCC |

(**a**) Red fond, catalytic core sequence of Dz8-17; PS linkage: s; OMe: underlined; LNA: Box. (**b**) Dz04-4s, Dz04-4OMe and Dz04-4LNA contained two PS, OMe and LNA modifications at both 5′ and 3′ ends of Dz04, respectively; Dz04-4s(2LNA)$_m$ contained one LNA in the middle of each Dz04-4s arm. Dz04-4s(2OMe)$_m$, Dz04-4s(4OMe)$_m$ and Dz04-4s(6OMe)$_m$ contained one, two and three OMe modifications in the middle of each Dz04-4s arm, respectively. (**c**) Dz4S-4s, Dz4S-4OMe and Dz4S-4LNA contained two PS, OMe and LNA modifications at both 5′ and 3′ ends of Dz4S, respectively. Dz4S-4s(2LNA)$_m$ contained one LNA in the middle of each Dz4S-4s arm. Dz4S-4s(2OMe)$_m$, Dz4S-4s(4OMe)$_m$ contained one and two OMe modifications in the middle of each Dz4S-4s arm, respectively. (**d**) Dz4M-4LNA contained two LNAs at both 5′ and 3′ ends of Dz4M.

**Table S4.** Dz04 sequences with various number of PS linkages on each arm.

| Dz8-17 | Sequence (5'-3')[a] |
|---|---|
| Dz04 | GGGGTAGCGGTCCGAGCCGGTCGAAGAAGCACTGCA |
| Dz04-2s[b] | GsGGGTAGCGGTCCGAGCCGGTCGAAGAAGCACTGCsA |
| Dz04-4s[b] | GsGsGGTAGCGGTCCGAGCCGGTCGAAGAAGCACTGsCsA |
| Dz04-6s[b] | GsGsGsGTAGCGGTCCGAGCCGGTCGAAGAAGCACTsGsCsA |
| Dz04-8s[b] | GsGsGsGsTAGCGGTCCGAGCCGGTCGAAGAAGCACsTsGsC sA |

(**a**) Catalytic core sequences of Dz8-17 are in red; PS: s. (**b**) Dz04 with one, two, three or four PS linkages at both ends.
